# Review of research progress in sepsis-associated acute kidney injury

**DOI:** 10.3389/fmolb.2025.1603392

**Published:** 2025-07-11

**Authors:** Wanning Nian, Weichen Tao, Haiyi Zhang

**Affiliations:** ^1^ Department of Health Management, Shengjing Hospital of China Medical University, ShenYang, China; ^2^ Department of Emergency Medicine, Shengjing Hospital of China Medical University, ShenYang, China

**Keywords:** sepsis, acute kidney injury (AKI), pathophysiological mechanisms, diagnosis, treatment, prognosis P2X7 receptor rat model, fecal peritonitis inhibition decreased renal IL-1β levels

## Abstract

Sepsis-associated acute kidney injury (SAAKI) poses a significant challenge in critical care medicine, characterized by high morbidity and mortality rates and often leading to chronic kidney disease (CKD). This article provides a comprehensive overview of the pathophysiological mechanisms, diagnostic advancements, therapeutic strategies, and prognostic studies of SAAKI. In terms of pathophysiological mechanisms, research has shifted from the traditional renal ischemia-centric view to a multidimensional interplay involving microcirculatory disturbances, immune metabolic disorders, and programmed cell death. Regarding diagnosis, traditional Kidney Disease: Improving Global Outcomes (KDIGO) criteria exhibit limitations, whereas novel biomarkers and imaging techniques offer new avenues for early diagnosis. Therapeutic strategies encompass early intervention, hemodynamic management, renal replacement therapy, and targeted therapies; however, controversy persists regarding the optimal timing and methods of their initiation. Prognostic studies focus on the mechanisms underlying the transition from SAAKI to CKD and corresponding preventive strategies. Future research should bridge the gap between animal models and human pathology and explore the potential of multi-omics technologies and artificial intelligence in optimizing management.

## Graphical Abstract

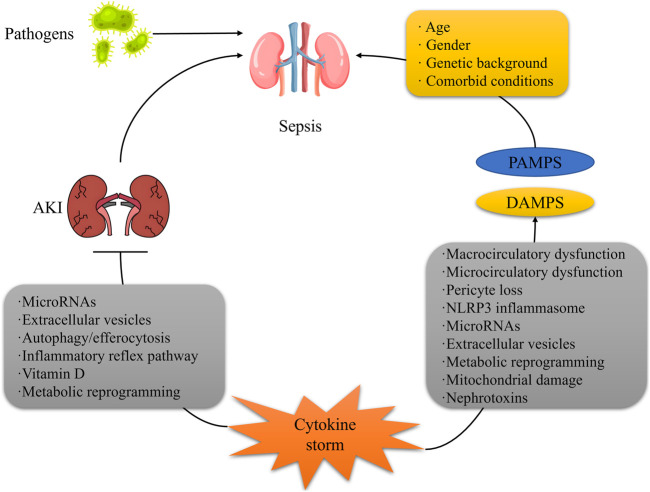

## 1 Introduction

Septic shock, the terminal phase of multiple organ dysfunction syndrome (MODS)induced by infection, is characterized by complex pathophysiological mechanisms, featuring circulatory failure and cellular metabolic derangements (Gavelli et al., 2021). Acute kidney injury (AKI), as one of the most prevalent complications of septic shock, is defined as septic shock-associated AKI (SA-AKI) (Poston and Koyner, 2019). In recent years, with the escalating incidence of sepsis globally, the clinical management of SA-AKI has emerged as a core challenge in critical care medicine (Zarbock et al., 2023a).

Globally, the annual incidence of sepsis is approximately 48.9 million cases (Font et al., 2020), with septic shock accounting for about 30%–50% of these cases. Notably, up to 60%–70% of septic shock patients develop AKI (Dugar et al., 2020). The mortality rate among SA-AKI patients is significantly higher than that of patients with sepsis or AKI alone, with in-hospital mortality rates reaching 40%–60%, and approximately 30% of survivors progressing to chronic kidney disease (CKD) (Zarbock et al., 2023b). Geographic variations are pronounced: in regions with limited medical resources (such as sub-Saharan Africa), the mortality rate of SA-AKI can reach 70%, whereas in high-income countries, early organ support can reduce mortality to 35%–40%. Notably, SA-AKI demonstrates a strong correlation with age, with elderly patients (>65 years) experiencing a 2.3-fold increase in incidence compared to younger patients due to decreased baseline renal function and immune senescence (Zarbock et al., 2023b; Pais et al., 2024).

SA-AKI accounts for 50%–60% of AKI cases in intensive care units (ICUs) and is a major factor contributing to prolonged ICU stays and soaring medical costs (Kounatidis et al., 2024). A multicenter cohort study (2023) indicated that the median ICU stay for SA-AKI patients was 12 days, 4.5 days longer than for non-SA-AKI septic shock patients, with *per capita* medical costs increasing by approximately $32,000 (White et al., 2023). Furthermore, SA-AKI is often accompanied by multi-organ failure, with about 45% of patients requiring renal replacement therapy (RRT), and 30% of these patients continuing to depend on RRT after discharge, further exacerbating the burden on the healthcare system (Wu X. et al., 2020). From a public health perspective, SA-AKI has become a significant component of the global “silent epidemic” of kidney diseases, and the World Health Organization (WHO) has prioritized it for chronic disease prevention and control by 2030 (Rippe, 2021; Domenichiello and Ramsden, 2019).

Despite the widespread recognition of the clinical importance of SA-AKI, its pathophysiological mechanisms remain controversial, and there is a lack of unified diagnostic and therapeutic standards (Fiorentino et al., 2024) ((Table 1). Traditional theories emphasize the central role of renal ischemic injury (Wu et al., 2022; Wang Y. et al., 2023), but recent research has found that renal injury in SA-AKI can occur under normal or even high renal blood flow conditions, suggesting the importance of non-hemodynamic mechanisms such as microcirculatory disturbances and immune metabolic disorders (Zhang et al., 2025; Prowle and Bellomo, 2015; Xu J. et al., 2023). Additionally, the emergence of novel biomarkers (e.g., NGAL, suPAR) and imaging techniques (e.g., renal ultrasound shear wave elastography) provides new tools for early diagnosis and prognosis assessment, although their clinical utility requires large-scale validation (Kounatidis et al., 2024; Xu J. et al., 2024; Leong et al., 2023; Gonçalves et al., 2022).

**TABLE 1:** Potential targets for diagnosis and treatment of sepsis-associated AKI found in preclinical studies.

Target	Model system	Intervention	Outcome	Reference
Microcirculatory dysfunction and pericyte loss				
Friend leukemia virus integration 1	Mouse, Cecal Ligation and Puncture (CLP) Model	Inhibition	Reduced Vascular Leakage, Improved Survival	Li et al. (2018)
miR-145a	Mouse Model	Inhibition	Increased Vascular Leakage, Decreased Survival	Wu et al. (2020b)
Platelet-Derived Growth Factor Receptor β (PDGFRβ)+ Pericytes	Swine Model, Lipopolysaccharide (LPS) Challenge	-	Enhanced Pericyte-to-Myofibroblast Transdifferentiation	Castellano et al. (2019)
NLRP3 inflammasome				
Sirtuin 3 (SIRT3)	Mouse Model, LPS Challenge	Inhibition	Increased Vascular Leakage, Decreased Survival	Zeng et al. (2016)
NLRP3	Mouse Model, CLP Model	Inhibition	Attenuated Kidney Injury	Cao et al. (2015)
Pannexin 1 (Panx1)	Mouse Model, LPS Challenge	Inhibition	Attenuated Kidney Injury	Huang et al. (2020)
P2X7 Receptor	Rat Model, Fecal Peritonitis	Inhibition	Decreased Renal IL-1β Levels	Arulkumaran et al. (2018)
MicroRNAs				
miR-452	Mouse Model, LPS/CLP Challenge		miR-452 ↑ before kidney injury	Liu et al. (2020a)
miR-762, miR-144–3p	Mouse Model, LPS Challenge		miR-762 ↑, miR-144–3p ↑	Tod et al. (2020)
miR-34–5b	Human LPS-Induced HK-2 Cells (*In Vitro*)	Inhibition/activation	Modulation of Inflammation and Apoptosis	Zheng et al. (2021)
Autophagy				
Autophagy-Related Gene 7 (Atg7)	Mouse Model, LPS Challenge	Inhibition	Exacerbated Kidney Injury	Mei et al. (2016)
Sirtuin 6 (SIRT6)	Human LPS-Induced HK-2 Cells (*In Vitro*)	Activation/inhibition	Modulation of Inflammation and Apoptosis	Zhang et al. (2019)
p53	Mouse Model, CLP Model	Deacetylation	Attenuated Kidney Injury	Sun et al. (2021)
Vitamin D Signaling				
Vitamin D Receptor (VDR)	Mouse Model, LPS Challenge	Inhibition/activation	Modulation of Kidney Injury	Du et al. (2019)
Vitamin D	Mouse Model, LPS Challenge	Deprivation	Exacerbated Kidney Injury	He et al. (2021)
Metabolic Reprogramming and Mitochondrial Function				
Peroxisome Proliferator-Activated Receptor Gamma Coactivator 1-Alpha (PGC-1α)	Mouse Model, LPS Challenge	Inhibition	Exacerbated Kidney Injury	Tran et al. (2011)
PTEN-Induced Putative Kinase 1 (PINK1)/Parkin RBR E3 Ubiquitin Protein Ligase (PARK2)	Mouse Model, LPS/CLP Challenge	Inhibition	Exacerbated Kidney Injury	Wang et al. (2021a)
Cyclic GMP-AMP Synthase (cGAS)	Mouse Model, LPS/CLP Challenge	Inhibition	Attenuated Kidney Injury	Visitchanakun et al. (2021)
Mitochondrial Reactive Oxygen Species (mtROS)	Mouse, CLP	Antioxidation by SS-31	Attenuated Kidney Injury	Li et al. (2016)
Dynamin-Related Protein 1 (Drp1)	Mouse Model, LPS Challenge	Inhibition by Mdivi-1	Attenuated Kidney Injury	Liu et al. (2020b)

Research on septic shock-associated AKI (SA-AKI) is shifting from traditional hemodynamic theories towards a paradigm focusing on the immune metabolic and programmed cell death axes (such as pyroptosis and ferroptosis). Mitochondrial autophagy reduces the release of damage-associated molecular patterns (DAMPs) from damaged mitochondria by clearing them, thereby inhibiting the activation of NLRP3 inflammasomes. Excessive activation of NLRP3 inflammasomes leads to cell pyroptosis and exacerbated inflammatory responses, while moderate autophagy helps to avoid this. At the diagnostic level, traditional KDIGO criteria have limitations, while breakthroughs in novel biomarkers and imaging techniques offer new means for early diagnosis and condition monitoring of SA-AKI. At the therapeutic level, clinical trials of individualized hemodynamic management and targeted anti-inflammatory drugs (such as recAP, IL-7 antagonists) provide new directions for the treatment of SA-AKI. At the prognostic level, research focuses on the molecular drivers and preventive strategies for the transition from SA-AKI to chronic kidney disease (CKD). By reviewing these advancements, this article attempts to answer the following key questions: Why do some SA-AKI patients respond poorly to traditional fluid resuscitation and vasoactive drugs? How can biomarkers be used for dynamic stratification to achieve precise renal replacement therapy (RRT) intervention? How should future research bridge the gap between animal models and human pathology, such as nephron heterogeneity? This article will also discuss the “know-do gap” in the diagnosis and treatment of SA-AKI based on updates from the 2021 Surviving Sepsis Campaign Guidelines (SSCG) and the 2023 KDIGO AKI Guidelines, and explore the potential of multi-omics technologies (single-cell sequencing, metabolomics) and artificial intelligence (predictive models, real-time monitoring) in optimizing SA-AKI management.

## 2 Progress in pathophysiological mechanisms of septic shock-associated acute kidney injury (SA-AKI)

The pathophysiological mechanisms of SA-AKI are intricate, involving multidimensional interactions such as hemodynamic derangements, immune-inflammatory storms, metabolic imbalances, and programmed cell death. Recent research has gradually transcended the traditional “renal ischemia-centric” paradigm, shifting towards an integrated perspective of microcirculation-immune-metabolic network regulation. The following sections systematically elaborate on these advancements from three aspects.

### 2.1 Hemodynamics and microcirculation impairment

#### 2.1.1 Controversy between renal perfusion pressure decline and vascular paralysis (traditional theory vs. new insights into microcirculation dysfunction)

**FIGURE 1:**
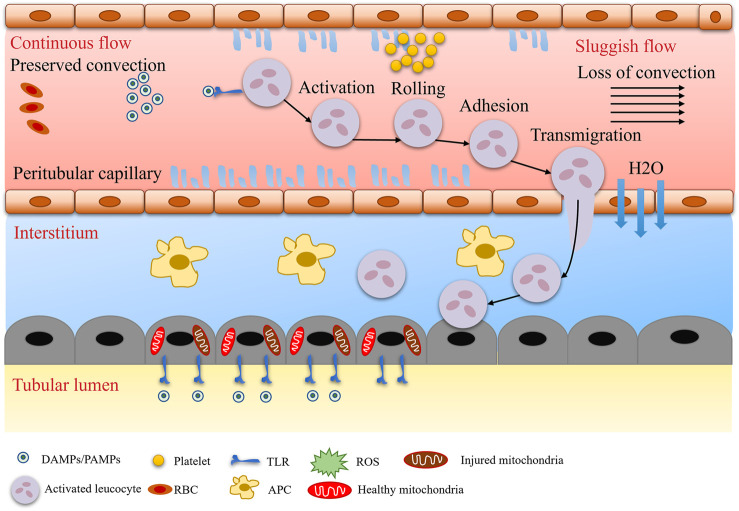
Microcirculatory and inflammatory alterations.

**FIGURE 2:**
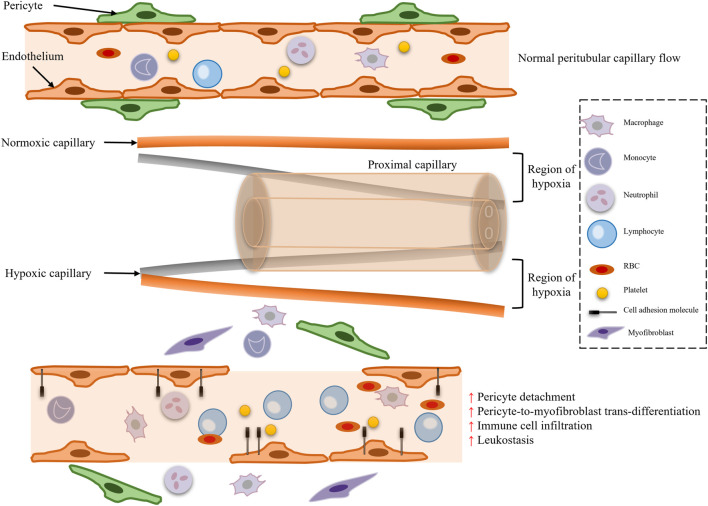
Microcirculatory dysfunction and pericyte loss.

Traditional theory posits that the core mechanism of SA-AKI is reduced renal blood flow due to systemic vasodilatation, known as the “renal ischemia hypothesis” (Figure 1). During septic shock, the systemic inflammatory response syndrome (SIRS) induces increased nitric oxide (NO) synthesis in vascular endothelial cells, leading to vasodilation, decreased blood pressure, and reduced renal perfusion pressure (RPP = mean arterial pressure - central venous pressure), subsequently triggering a decline in glomerular filtration rate (GFR) (Figure 2). Animal model evidence shows a 50%–70% reduction in renal blood flow in septic rats, negatively correlating with serum creatinine elevation (Langenberg et al., 2006a; Langenberg et al., 2006b). Clinical interventions also indicate that early fluid resuscitation combined with norepinephrine to elevate mean arterial pressure (MAP) can partially restore urine output (Hernández et al., 2019). However, recent studies have found that renal blood flow does not significantly decrease in some SA-AKI patients, and even a “hyperemic AKI” phenomenon exists, focusing attention on microcirculation dysfunction as a novel mechanism. Microcirculatory shunting manifests as uneven distribution of capillary blood flow within the kidney, with stasis in some regions while large vessel blood flow remains normal (Ospina-Tascón et al., 2020). The paradox of oxygen metabolism suggests increased renal venous oxygen saturation (RvO_2_), reflecting impaired oxygen utilization rather than simple oxygen delivery deficiency (Prowle, 2014). The specific manifestation of vascular paralysis is the reduced responsiveness of renal vessels to catecholamines, potentially linked to mitochondrial reactive oxygen species (ROS) inhibiting calcium-sensitive proteins in vascular smooth muscle cells (e.g., Rho kinase) (Milich et al., 2021). The current controversy centers on whether the traditional theory can explain all SA-AKI subtypes, with hemodynamic mechanisms dominating in early stages (<6 h) and microcirculation dysfunction playing a crucial role in sustained injury, necessitating stratified analysis based on patient endotypes (Stevens et al., 2024).

#### 2.1.2 Crucial role of endothelial cell injury and glycocalyx shedding

Renal microvascular endothelial injury is one of the initiating factors in SA-AKI, with glycocalyx degradation as its core manifestation (Xing et al., 2023; Molema et al., 2022). The glycocalyx, a proteoglycan layer (e.g., syndecan-1, hyaluronic acid) covering the endothelial cell surface, maintains vascular permeability and anticoagulant activity (Foote et al., 2022; Tarbell and Cancel, 2016). During sepsis, matrix metalloproteinase-9 (MMP-9) and hyaluronidase (HYAL1) activity upregulates, directly cleaving the glycocalyx (Xing et al., 2023; Lin et al., 2024); TNF-α and IL-1β inhibit glycocalyx synthase (e.g., EXTL3) via the TLR4/NF-κB pathway (Lei et al., 2021; Marques et al., 2022). Glycocalyx shedding leads to a series of pathological consequences: capillary leakage results in albumin extravasation and interstitial edema compressing renal tubules (Rehm et al., 2004; Gamez et al., 2024); loss of the endothelial anticoagulant barrier activates the coagulation cascade (e.g., vWF release), exacerbating intrarenal thrombotic microangiopathy (TMA) (Kei et al., 2023; Peter et al., 2023; Medica et al., 2024); and exposed adhesion molecules (e.g., ICAM-1) promote neutrophil infiltration, amplifying inflammatory damage (Zhang W. et al., 2020). Experimental glycocalyx protectants (e.g., sucrose octasulfate, anti-HYAL1 antibodies) mitigate AKI in sepsis models but have yet to enter clinical translation (Xing et al., 2023; Zhang D. et al., 2020; Yu et al., 2019).

### 2.2 Immune and inflammatory responses

#### 2.2.1 Mechanisms of pyroptosis and neutrophil extracellular traps (NETs)

Pyroptosis, mediated by Gasdermin D (GSDMD), is an inflammatory programmed cell death modality playing a crucial role in SA-AKI (Wu et al., 2022). In a septic environment, pathogen-associated molecular patterns (PAMPs, e.g., LPS) bind to Toll-like receptor 4 (TLR4) on the cell surface, activating the intracellular NLRP3 inflammasome. Activated NLRP3 inflammasome promotes caspase-1 cleavage, which in turn cleaves GSDMD. The N-terminal fragment of GSDMD forms pores in the cell membrane, increasing cell permeability, leading to the release of cellular contents and promoting the release of interleukin-1β (IL-1β) and interleukin-18 (IL-18), triggering a robust inflammatory response (Li T. et al., 2022).

Deng Y et al. further investigated the role of pyroptosis in SA-AKI. They found that GSDMD expression was significantly upregulated in tubular epithelial cells (TECs), positively correlating with AKI severity. In gene knockout mouse models, knocking out the GSDMD gene significantly improved renal function, decreased renal injury marker levels, and mitigated renal histopathological damage in sepsis-induced AKI models (Jiang et al., 2021; Deng et al., 2021). This suggests that GSDMD-mediated pyroptosis plays a key role in the onset and progression of SA-AKI, and inhibiting pyroptosis may become a potential therapeutic strategy. NETs are chromatin web structures released by neutrophils, primarily capturing and killing pathogens. However, excessive NETosis leads to tissue damage in pathological states such as sepsis. Keshari A et al. studied the toxicity of NETs components. They found that components such as histones (e.g., H3Cit) and myeloperoxidase (MPO) in NETs directly induce TEC apoptosis. These components bind to receptors on the TEC surface, activating intracellular apoptotic signaling pathways, leading to cell death (Ni et al., 2021). Some studies have focused on the role of NETs in microcirculation. They found that NETs can aggregate with platelets to form thrombi, blocking renal capillaries. This microcirculatory obstruction causes local renal ischemia, further exacerbating renal injury (Ni et al., 2021). Additionally, NETs formation activates the coagulation system, promoting microthrombosis and aggravating renal pathological damage (Wu et al., 2023).

To intervene in NETs-mediated renal injury, researchers have explored multiple potential therapeutic targets. DNase I, an enzyme that degrades DNA, can effectively degrade NETs chromatin, thereby mitigating NETs-induced renal damage (Raup-Konsavage et al., 2018; Wang H. et al., 2023). Furthermore, PAD4 inhibitors (e.g., GSK484) can also exert renal protective effects by inhibiting NETs formation (Wang B. et al., 2023; Perdomo et al., 2019). These interventions have shown promising renal protective effects in animal models, providing new avenues for future clinical treatment of SA-AKI.

#### 2.2.2 Mitochondrial dysfunction and metabolic reprogramming

**FIGURE 3:**
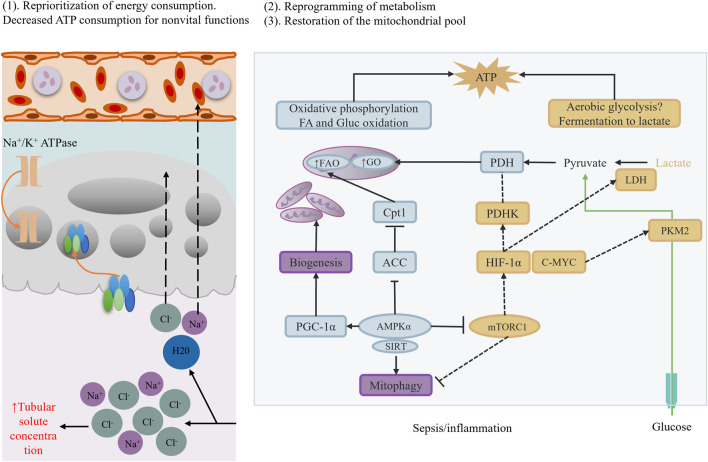
Metabolic reprogramming.

Mitochondria, the energy factories of cells, play a crucial role in SA-AKI. Studies have found that renal cell mitochondrial dynamics are imbalanced during sepsis, manifesting as abnormalities in mitochondrial fusion and fission processes (Pais et al., 2024; Chen Y. et al., 2024) (Figure 3). Additionally, mitochondrial oxidative phosphorylation (OXPHOS) is inhibited, leading to energy metabolic disorders (Yu et al., 2018). Specifically, mitochondrial complex I dysfunction results in excessive superoxide production, which can activate the NLRP3 inflammasome, further exacerbating inflammation (Billingham et al., 2022). Damaged mitochondria release mitochondrial DNA (mtDNA) into the cytoplasm. As a damage-associated molecular pattern (DAMP), mtDNA can activate the type I interferon response via the cGAS-STING pathway, leading to overactivation of the immune system and exacerbating renal injury (Amador-Martínez et al., 2023).

During sepsis, renal cell metabolic processes undergo significant reprogramming. Sepsis downregulates the expression of peroxisome proliferator-activated receptor α (PPARα) (Vandewalle and Libert, 2022). PPARα is a key metabolic regulator, and its downregulation inhibits fatty acid oxidation (FAO) (Chen et al., 2022). Specifically, PPARα downregulation reduces the expression of carnitine palmitoyltransferase 1A (CPT1A), a key enzyme for fatty acids to enter mitochondria for oxidation (Dong J. et al., 2024). Therefore, downregulation of PPARα and CPT1A leads to fatty acid accumulation within cells, forming lipotoxicity and further damaging cells. Simultaneously, renal cell metabolic processes compensatorily enhance glycolysis. Studies have found that hypoxia-inducible factor-1α (HIF-1α) is activated during sepsis, promoting glucose uptake (Liu et al., 2023). However, TECs lack hexokinase 2 (HK2), rendering glucose ineffective in converting to energy, leading to insufficient ATP synthesis within cells. This imbalance in energy metabolism further exacerbates cell damage and dysfunction (Shu et al., 2024).

Based on these mechanistic studies, potential therapeutic strategies are being explored. For example, PPARα agonists (e.g., fenofibrate) can restore fatty acid oxidation, mitigate lipotoxicity, and improve cellular energy metabolic status (Hong et al., 2014; Castro et al., 2024; Tang et al., 2023)]. Additionally, mitochondrial antioxidants (e.g., SS-31) can protect mitochondrial function by scavenging reactive oxygen species (ROS) within mitochondria, mitigating oxidative stress damage (Zheng et al., 2022; Peng et al., 2021). However, despite these important research advancements, many critical questions remain to be addressed. For instance, SA-AKI mechanisms significantly differ across disease stages (early vs. sustained) and renal zones (cortex vs. medulla), posing a challenge for precision interventions targeting these spatiotemporal heterogeneities. Moreover, current animal models cannot fully mimic the complex pathological processes of human SA-AKI, limiting clinical translation of research findings. In the future, emerging technologies such as single-cell spatial transcriptomics, organoid models, and dynamic biosensing technologies are expected to reveal real-time mechanistic maps of SA-AKI, revolutionizing targeted therapies.

In summary, pyroptosis, NETs-mediated damage, mitochondrial dysfunction, and metabolic reprogramming play crucial roles in the onset and progression of SA-AKI. A deeper understanding of these mechanisms will provide a theoretical basis for developing novel therapeutic strategies, potentially improving the prognosis of SA-AKI patients.

### 2.3 Novel mechanisms of tubular injury

#### 2.3.1 Sublethal injury and cell cycle arrest

Sublethal injury refers to TECs not fully dying but losing differentiation and repair capabilities under septic stimuli.

##### 2.3.1.1 Hallmark features

(1) Loss of Cell Polarity: In sepsis-induced tubular injury, TECs exhibit loss of cell polarity, such as mislocalization of Na+/K + ATPase. This polarity loss implies disruption of normal cell function and structure, unable to maintain normal ion balance and cell morphology (Wang D. et al., 2023; Cantaluppi et al., 2018).
(2) Brush Border Shedding: The brush border, a vital functional structure of TECs, is responsible for absorption and transport of substances. Under septic stimuli, brush border shedding impairs the absorption and transport functions of cells (Wang D. et al., 2023).
(3) Preservation of Cell Membrane Integrity: Despite polarity loss and brush border shedding, cell membrane integrity is preserved. This means cells have not completely died but have lost normal physiological functions, residing in a sublethal state (Wang D. et al., 2023).

##### 2.3.1.2 Molecular mechanisms

(1) Activation of the DNA Damage Response (DDR) p53/p21 Pathway: Inflammation and oxidative stress induced by sepsis can cause DNA damage in TECs. This damage activates the DDR pathway, subsequently activating the p53/p21 pathway, inducing cell cycle arrest at the G1/S phase (Yang et al., 2022; Qi et al., 2021). p53 is a crucial tumor suppressor gene, whose activation leads to cell cycle arrest, preventing damaged cells from entering the cell cycle and thus avoiding potential genetic mutations and cellular carcinogenesis.
(2) Cell Cycle Arrest: Following DDR activation, the cell cycle arrests at the G1/S phase, preventing cells from entering the S phase for DNA synthesis and cell division. This arrested state impedes normal repair and regeneration, leading to long-term impairment of cell function (Kumari and Jat, 2021).

##### 2.3.1.3 Clinical implications

(1) Secretion of Senescence-Associated Secretory Phenotype (SASP): TECs in a sublethal injury state secrete SASP, including cytokines such as IL-6 and TGF-β (Schroth et al., 2020; Xie et al., 2024). These cytokines have pro-inflammatory and pro-fibrotic effects, further exacerbating renal inflammation and fibrosis (Yin et al., 2024).
(2) Promotion of Fibrotic Microenvironment: SASP secretion not only affects damaged cells but also influences surrounding cells and tissues, promoting the formation of a fibrotic microenvironment. This microenvironment favors the deposition of extracellular matrix and activation of fibroblasts, thereby exacerbating renal fibrosis and leading to continuous decline in renal function (Li et al., 2021a; Bhalla et al., 2022).

##### 2.3.1.4 Intervention strategies

###### 2.3.1.4.1 Senolytics drugs.

Senolytics are drugs that selectively eliminate senescent cells (Zhang et al., 2023a). Combined use of dasatinib and quercetin has been shown to effectively clear senescent TECs (Hickson et al., 2019). In SA-AKI models, this intervention strategy significantly improved renal pathological damage and functional recovery, providing new insights for clinical treatment (Rao et al., 2024; Zhou S. et al., 2024).

#### 2.3.2 Molecular evidence of autophagy dysregulation

##### 2.3.2.1 Protective phase

###### 2.3.2.1.1 Moderate autophagy clears damaged mitochondria.

**FIGURE 4:**
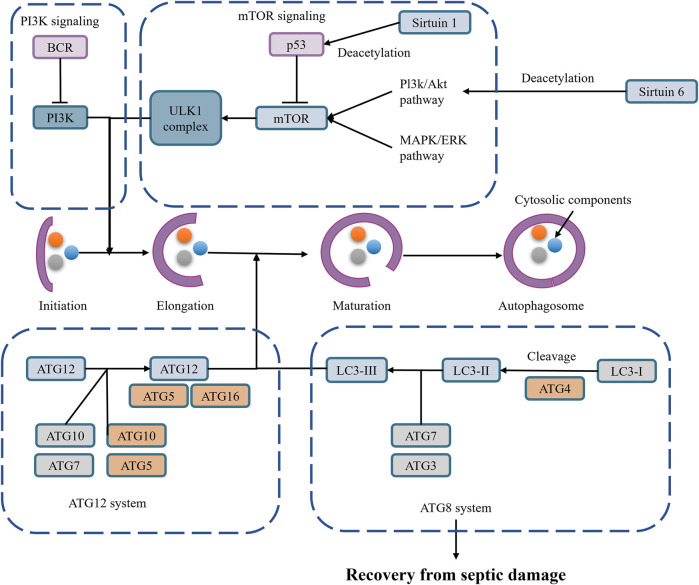
Autophagy in SAAKI.

In the early stages of sepsis, moderate autophagy can clear damaged mitochondria, maintaining cellular energy metabolism and function through mitophagy. This autophagy process helps inhibit NLRP3 inflammasome activation, reducing inflammation and protecting cells (Wang D. et al., 2023; Leventhal et al., 2016; Toro et al., 2021) (Figure 4).

Mitophagy, a selective autophagy, eliminates damaged and excess mitochondria, thereby regulating NLRP3 inflammasome activity (Jiang et al., 2020; Su et al., 2023). Damaged mitochondria release damage-associated molecular patterns (DAMPs), such as mitochondrial DNA and mitochondrial reactive oxygen species (mtROS), which are important signals for NLRP3 inflammasome activation (Lin et al., 2019). Mitophagy reduces the release of these DAMPs, thereby inhibiting NLRP3 inflammasome activation.

**FIGURE 5:**
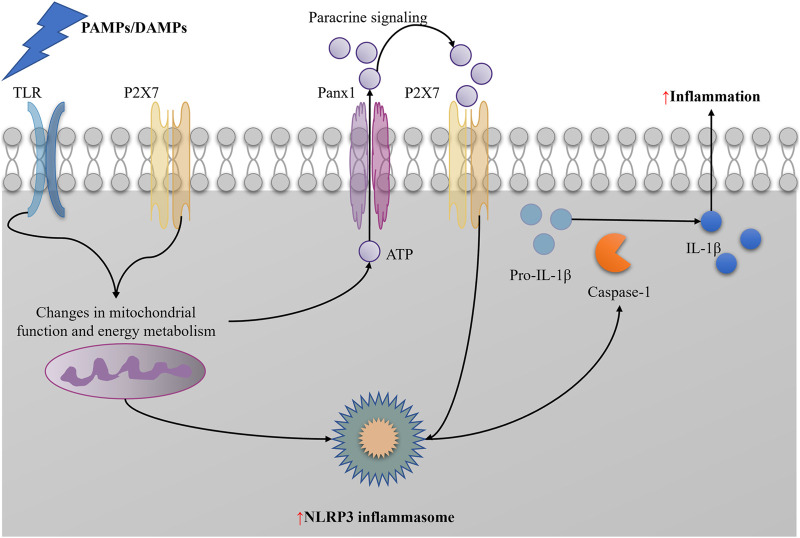
NLRP3 induce inflammation in SAAKI.

NLRP3 inflammasome activation may provide feedback regulation on mitophagy. On the one hand, overactivation of the NLRP3 inflammasome may further exacerbate mitochondrial damage, increasing the demand for mitophagy (Lin et al., 2021); on the other hand, inflammatory factors produced after NLRP3 inflammasome activation may affect intracellular metabolic status and signaling pathways, indirectly regulating mitophagy levels (Lin et al., 2021). NLRP3 inflammasome activation is a crucial factor in pyroptosis. Activated NLRP3 inflammasome recruits and activates caspase-1, which then cleaves Gasdermin D (GSDMD), forming membrane pores, leading to cell swelling, rupture, and eventual pyroptosis. The pyroptosis process releases a large amount of inflammatory factors, further exacerbating inflammation (Guo et al., 2024). Besides directly causing cell pyroptosis, NLRP3 inflammasome activation promotes the maturation and secretion of multiple inflammatory factors, such as interleukin-1β (IL-1β) and interleukin-18 (IL-18), which play crucial roles locally and systemically, triggering and exacerbating inflammation (Wang et al., 2024; Sun et al., 2022) (Figure 5).

###### 2.3.2.1.2 Inhibition of NLRP3 activation.

Mitophagy inhibits NLRP3 inflammasome activation by clearing damaged mitochondria and reducing the release of DAMPs. Overactivation of the NLRP3 inflammasome leads to pyroptosis and exacerbated inflammation, and the protective effect of moderate autophagy helps avoid these scenarios (Wang D. et al., 2023; Leventhal et al., 2016; Toro et al., 2021). Moderate autophagy can eliminate harmful substances within cells, such as damaged mitochondria and protein aggregates, maintaining intracellular environmental stability and thereby protecting cells. In the context of NLRP3 inflammasome activation, moderate autophagy inhibits the overactivation of the NLRP3 inflammasome by clearing damaged mitochondria and reducing the release of DAMPs, thus preventing pyroptosis and inflammation (Piantadosi and Suliman, 2012; Fu et al., 2023; Oh and Lee, 2014). Autophagy can regulate NLRP3 inflammasome activation through multiple pathways. On the one hand, autophagy-related proteins such as Beclin-1 participate in regulating mitophagy, thereby affecting mitochondrial ROS production and NLRP3 inflammasome activation (Zhao et al., 2021); on the other hand, autophagy can degrade NLRP3 protein itself or its upstream regulators to inhibit NLRP3 inflammasome activation. For example, ABHD8 recruits palmitoyltransferase ZDHHC12 to bind with NLRP3, mediating NLRP3 palmitoylation modification, enhancing NLRP3 binding with the molecular chaperone HSC70, thereby promoting NLRP3 degradation through chaperone-mediated autophagy (Spalinger et al., 2020).

In-depth study of the relationship between mitophagy and NLRP3 inflammasome activation can help discover new therapeutic targets. For instance, by regulating mitophagy or directly intervening in NLRP3 inflammasome activation, it is possible to develop new therapies for inflammatory diseases and autoimmune diseases.

##### 2.3.2.2 Dysfunctional phase

(1) Lysosomal Acidification and ATG5 Degradation: In the late stages of sepsis, lysosomal acidification and ATG5 degradation lead to autophagy flux blockade (Suzuki et al., 2023; Huang et al., 2023). ATG5 is a crucial protein in the autophagy process, and its degradation blocks normal autophagy, leading to accumulation of autophagy intermediates.
(2) p62 Activates the Keap1/Nrf2 Oxidative Stress Pathway: Autophagy flux blockade results in p62 protein accumulation, which can activate the Keap1/Nrf2 oxidative stress pathway (Zhai et al., 2024; Liao et al., 2019). Nrf2 is an important antioxidant transcription factor, and its activation promotes the expression of antioxidant genes. However, excessive oxidative stress responses still cause cellular damage.

#### 2.3.3 Ferroptosis mechanism

##### 2.3.3.1 Lipid peroxidation drive

(1) ACSL4-Mediated Phospholipid Esterification: Ferroptosis is a cell death mode driven by iron-catalyzed lipid peroxidation. In septic conditions, ACSL4 protein mediates the esterification of polyunsaturated fatty acids (PUFAs) into phospholipids, providing substrates for lipid peroxidation (Wu et al., 2024; Lai et al., 2023; Xiao et al., 2024).
(2) LOXs-Catalyzed Oxidation: LOXs (lipoxygenases) catalyze the oxidation of PUFAs, generating lipid peroxides. These peroxides accumulate in cell membranes, causing membrane damage and dysfunction (Wang B. et al., 2021).
(3) Insufficient GPX4 Activity: Glutathione peroxidase 4 (GPX4) is a key regulator of ferroptosis, and its insufficient activity leads to the accumulation of lipid peroxides, ultimately leading to loss of cell membrane integrity (Zan et al., 2024; Li J. et al., 2023).

##### 2.3.3.2 Renal-specific evidence

(1) Elevated Urinary Lipid Peroxide Levels: In SA-AKI patients, urinary lipid peroxide levels (e.g., MDA, 4HNE) are significantly elevated. These peroxide levels negatively correlate with GPX4 expression, indicating that ferroptosis plays an important role in SA-AKI (Zhang et al., 2024).
(2) Therapeutic Exploration: Iron chelators (e.g., deferoxamine) and GPX4 activators (e.g., RSL3) have shown efficacy in mitigating tubular injury in septic mouse models (Zhang et al., 2023b; Liu et al., 2025). These drugs inhibit lipid peroxidation by reducing iron accumulation and enhancing GPX4 activity, thereby mitigating ferroptosis-induced renal damage.

#### 2.3.4 Progress and future directions in SA-AKI mechanisms

Recent studies on SA-AKI mechanisms have shifted from a single hemodynamic model to a multidimensional interactive network. However, several critical issues remain to be addressed.

##### 2.3.4.1 Spatiotemporal heterogeneity

Differences in Mechanisms Across Disease Stages: In the early stages (<6 h) of SA-AKI, hemodynamic mechanisms may dominate. However, in sustained injury stages, the roles of microcirculation dysfunction and intracellular molecular mechanisms gradually emerge (Wang D. et al., 2023). Therefore, it is necessary to develop tailored treatment strategies based on the mechanistic characteristics of different disease stages.

Differences in Renal Zones: The injury mechanisms also vary across different renal zones (e.g., cortex and medulla) in SA-AKI. For instance, the medulla is more susceptible to ischemia and inflammation due to its unique anatomical structure and physiological functions. Therefore, further study of injury mechanisms in different renal zones is needed to achieve precision treatment (Wang D. et al., 2023).

#### 2.3.5 Translational bottlenecks

##### 2.3.5.1 Limitations of animal models

Current animal models cannot fully mimic the complex pathological processes of human SA-AKI. For example, mice lack medullary straight vessels, posing limitations in studying medullary injury mechanisms in animal models. Therefore, it is necessary to develop animal models closer to human pathological processes or utilize new technologies such as organoid models to better simulate and study SA-AKI mechanisms.

#### 2.3.6 Precision interventions

(1) Application of Biomarkers: How to distinguish different SA-AKI endotypes (e.g., inflammation-dominant vs. metabolic disorder) using biomarkers is an urgent issue. The discovery and application of biomarkers will facilitate individualized treatment and improve treatment outcomes (Manrique-Caballero et al., 2021; Kuwabara et al., 2022).
(2) Individualized Treatment Strategies: Formulating individualized treatment plans based on patients’ specific pathological mechanisms and endotypes is an important direction for future SA-AKI treatment (Manrique-Caballero et al., 2021; Kuwabara et al., 2022). For example, anti-inflammatory treatment can be adopted for inflammation-dominant SA-AKI patients, while metabolic regulation therapy can be considered for metabolic disorder patients.

In the future, with the development of emerging technologies such as single-cell spatial transcriptomics, organoid models, and dynamic biosensing technologies, it is expected to reveal real-time mechanistic maps of SA-AKI, providing more precise evidence for targeted therapy. These technologies will help us better understand the complex pathological processes of SA-AKI, driving innovation and development in treatment strategies.

## 3 Diagnosis and biomarkers

### 3.1 Limitations of traditional diagnostic criteria

The Kidney Disease: Improving Global Outcomes (KDIGO) criteria, which are extensively utilized in clinical practice for the diagnosis of acute kidney injury (AKI), primarily encompass alterations in serum creatinine concentration and urine output (Levey, 2022; Meersch et al., 2017). However, the application of KDIGO criteria in SAAKI is fraught with limitations:

Creatinine Lag Effect: The change in serum creatinine concentration often lags behind the actual renal functional impairment. Following a decline in glomerular filtration rate (GFR), it may take 48–72 h for creatinine concentrations to appreciably elevate (Szumilas et al., 2024). This lag effect obstructs the timely detection of early renal functional damage, particularly pronounced in elderly patients or those with reduced muscle mass.

Interfering Factors in Urine Output: Urine output serves as a pivotal indicator in KDIGO criteria but is susceptible to numerous interfering factors in practical application (Ji et al., 2022). For instance, in septic patients, urine output may be influenced by interventions such as diuretics and continuous renal replacement therapy (CRRT). Additionally, patients with non-oliguric AKI may exhibit normal urine output despite the presence of renal functional impairment (Szumilas et al., 2024).

### 3.2 Emerging biomarkers

#### 3.2.1 Injury biomarkers: clinical validation of NGAL, KIM1, and IL18

With the in-depth exploration of the pathophysiological mechanisms underlying AKI, several novel biomarkers have been identified and introduced into clinical practice. Among them, neutrophil gelatinase-associated lipocalin (NGAL), kidney injury molecule-1 (KIM-1), and interleukin-18 (IL-18) exhibit the greatest potential (Szumilas et al., 2024; Goldstein, 2015).

NGAL: NGAL, a 25 kD protein primarily stored in neutrophil granules and expressed in various tissues, undergoes marked upregulation upon renal injury, detectable through urine and blood tests Sun et al. (2021). Studies have demonstrated that NGAL can indicate the onset of AKI in septic patients with higher sensitivity and specificity than serum creatinine (Sun et al., 2024).

KIM-1: KIM-1, a transmembrane glycoprotein, undergoes substantial upregulation in response to renal ischemic or toxic injury. By clearing apoptotic cellular debris and oxidized lipids within the lumen, KIM-1 mitigates renal damage (Alhilal et al., 2024). Clinical studies have closely associated changes in urine KIM-1 concentrations with the occurrence and severity of AKI (Brozat et al., 2024).

IL-18: IL-18, a pro-inflammatory cytokine, is increasingly recognized for its role in renal injury. Research has shown that urine IL-18 concentrations can significantly elevate prior to AKI onset, enabling early prediction of AKI (Zhou J. et al., 2024). Furthermore, IL-18 has proven useful in identifying severe tubular necrosis in renal transplant patients (Liu et al., 2018; Piancatelli et al., 2016).

#### 3.2.2 Functional biomarkers: comparative advantages of cystatin C vs. creatinine

Cystatin C, a low-molecular-weight protein produced by nuclear cells and almost exclusively filtered by the glomerulus for subsequent reabsorption and degradation (Shlipak et al., 2022; Ding et al., 2022), offers several advantages over serum creatinine:

Minimal Influence from Non-Renal Factors: Cystatin C concentrations are less affected by factors such as age, gender, and muscle mass, thus more accurately reflecting GFR (Xu X. et al., 2023; Farrington et al., 2023).

Higher Early Sensitivity: Cystatin C exhibits greater sensitivity in detecting early renal functional impairment, enabling earlier identification of renal abnormalities (Campbell et al., 2023).

#### 3.2.3 Omics technology breakthroughs: urinary exosomal miRNAs and metabolomic profiles

With advancements in omics technology, urinary exosomal miRNAs and metabolomic profiles have emerged as research hotspots (Erdbrügger et al., 2021; McGlinchey et al., 2022). Urinary exosomal miRNAs reflect renal cellular injury status, offering potential for early diagnosis and prognostic assessment (Dong Y. J. et al., 2024; Lu et al., 2020). Metabolomic technology, by analyzing urine metabolite profiles, reveals metabolic characteristics of renal injury, providing novel insights for early AKI diagnosis and treatment (Kammer et al., 2019).

### 3.3 Radiological progress

Bedside ultrasonography, a non-invasive and convenient imaging technique, assesses renal blood flow perfusion. Renal resistive index (RRI), derived from ultrasound measurement of renal arterial blood flow resistance, reflects renal blood flow perfusion status. Studies have shown a close correlation between elevated RRI and AKI occurrence and severity, underscoring its value in early AKI prediction (Shen et al., 2023; Barone et al., 2024)。.

Functional MRI techniques, such as blood oxygen level-dependent (BOLD) imaging, provide renal oxygenation and blood flow perfusion information (Hobson et al., 2017). By detecting the ratio of oxygenated to deoxygenated hemoglobin in renal tissue, BOLD imaging reflects renal oxygenation status (Hobson et al., 2017). This technology holds potential for early detection of renal ischemia and injury but remains in the research phase (Li A. et al., 2022; Bauer et al., 2017).

## 4 Optimization of therapeutic strategies

### 4.1 Early intervention and prevention

#### 4.1.1 Impact of the golden hour bundle therapy on renal prognosis

Early intervention is crucial in sepsis and septic shock management, particularly within the “golden hour” for bundle therapy (Bissell et al., 2019; Rhodes et al., 2017). Bundle therapy combines a series of proven effective treatment measures to enhance therapeutic efficacy and patient prognosis. For renal function protection, early fluid resuscitation and hemodynamic management are pivotal (Saad and Maybauer, 2017).

Rapid administration of 30 ml/kg of crystalloid for fluid resuscitation is a vital component of early intervention in septic shock patients. This measure aims to swiftly restore blood volume, maintain effective tissue perfusion and oxygenation, thereby reducing AKI risk. However, recent guidelines have downgraded this recommendation from “recommended” to “suggested,” indicating the need for individualized adjustment based on patient-specific conditions (Aston et al., 2023).

#### 4.1.2 Restrictive fluid resuscitation and choice of balanced crystalloids (e.g., ringer’s acetate vs. normal saline)

Fluid resuscitation is essential in sepsis treatment, but excessive fluid infusion may lead to interstitial edema, dilutional coagulopathy, and immune dysfunction. Consequently, the restrictive fluid resuscitation strategy has garnered attention. Studies have shown that restrictive fluid resuscitation reduces mechanical ventilation duration and improves prognosis without increasing AKI or renal replacement therapy (RRT) risk.

Differences exist between restrictive and traditional fluid resuscitation in sepsis treatment outcomes. In terms of 60-day mortality, the early goal-directed therapy (EGDT) group exhibited significantly lower mortality than the standard care group (Investigators et al., 2014), though some studies found no significant improvement in 60-day mortality in the EGDT group (Mouncey Paul et al., 2015). For 90-day mortality, the CLASSIC trial found no mortality reduction benefit with restrictive fluid resuscitation compared to standard fluid resuscitation (Plummer and Bellomo, 2022). In terms of organ support needs, the EGDT group was significantly lower than the standard care group (Investigators et al., 2014), but no significant difference was observed between the restrictive and standard fluid resuscitation groups in the CLASSIC trial (Plummer and Bellomo, 2022). For hospital stay, the EGDT group was significantly longer than the standard care group (Investigators et al., 2014), while no significant difference was found between the two groups in the CLASSIC trial (Plummer and Bellomo, 2022). In terms of vasoactive drugs and red blood cell transfusions, the EGDT group showed a significant increase in usage (Mouncey Paul et al., 2015), but no significant difference was observed between the two groups in the CLASSIC trial (Plummer and Bellomo, 2022). In terms of microcirculation and hemodynamic indicators, the restrictive fluid resuscitation strategy places greater emphasis on hemodynamic stability and microcirculation improvement (Bakker et al., 2022; Wheeler, 2015). In terms of renal replacement therapy (RRT) usage, no significant difference was observed between the two groups in the CLASSIC trial (Plummer and Bellomo, 2022). In terms of long-term survival, a multicenter trial showed significantly higher long-term survival in the restrictive fluid resuscitation group compared to the standard care group, but no significant difference was observed between the two groups in the CLASSIC trial. In terms of adverse events, the incidence of serious adverse events in the restrictive fluid resuscitation group was not significantly different from that in the standard care group.

Regarding fluid choice, balanced crystalloids (e.g., lactate Ringer’s solution or Ringer’s acetate solution) offer certain advantages over normal saline. Normal saline may induce hyperchloremic metabolic acidosis, whereas balanced crystalloids better maintain electrolyte balance and reduce renal burden. Additionally, for patients receiving substantial crystalloid resuscitation, combining albumin is recommended to enhance plasma colloid osmotic pressure and mitigate tissue edema.

### 4.2 Hemodynamic management

#### 4.2.1 Vasopressor selection: renal protective effects of norepinephrine combined with vasopressin

In septic shock treatment, vasopressor use is crucial for maintaining blood pressure and tissue perfusion. Norepinephrine is the preferred vasopressor due to its effectiveness in increasing mean arterial pressure (MAP) with minimal impact on cardiac output. However, norepinephrine alone may insufficiently maintain adequate renal perfusion, prompting the common strategy of combining vasopressin.

The use of norepinephrine combined with low-dose vasopressin in septic shock treatment presents potential side effects. On the one hand, low-dose vasopressin may reduce the risk of renal failure, as demonstrated by the VANISH trial (Gordon et al., 2016), but other studies have indicated that it may cause renal impairment (Huang et al., 2021). On the other hand, vasopressin maintains MAP by increasing vascular resistance, which may lead to hypertension, negative effect especially in patients with hypertension or cardiovascular disease risks (Nagendran et al., 2019). Furthermore, its use may also cause adverse reactions such as myocardial ischemia and intestinal ischemia, resulting in hyponatremia, affecting the nervous system and overall health, and increasing the risk of certain adverse reactions when used in combination with corticosteroids, such as hypertension and renal impairment (Nagendran et al., 2019). Additionally, vasopressin may cause other adverse reactions such as skin necrosis and decreased platelet count (Morelli et al., 2009).

#### 4.2.2 Individualized MAP targets (precision management based on microcirculation monitoring)

Traditional hemodynamic management primarily focuses on systemic hemodynamic parameters such as MAP and central venous pressure (CVP). However, these parameters do not fully reflect tissue and organ perfusion status. Recently, advancements in microcirculation monitoring technology have provided new means for individualized hemodynamic management.

By monitoring microcirculation, a more accurate assessment of tissue perfusion status can be achieved, enabling individualized MAP target management. For example, some patients may require a higher MAP to ensure renal perfusion, while for others, a lower MAP may be sufficient. This precision management strategy helps reduce the incidence of renal injury and improve patient prognosis.

### 4.3 Controversies in renal replacement therapy (CRRT)

#### 4.3.1 Timing of initiation: Traditional criteria vs. insights from the ELAIN/IDEAL-ICU trials

CRRT is a vital means of treating severe AKI, but the timing of its initiation has been a controversial issue in clinical practice. Traditionally, the timing of CRRT initiation has been based on changes in serum creatinine levels and urine output. However, recent studies suggest that early initiation of CRRT may be more beneficial to patient prognosis.

The ELAIN and IDEAL-ICU trials provide new insights into the timing of CRRT initiation (Tomar et al., 2021; Gaudry et al., 2022; Zarbock et al., 2016). Both the ELAIN trial and the IDEAL-ICU trial explored the timing of initiating continuous renal replacement therapy (CRRT) in patients with AKI. The ELAIN trial enrolled 231 patients meeting KDIGO Stage 2 AKI criteria and found that early initiation of CRRT (within 8 h after diagnosis) significantly reduced 90-day all-cause mortality (39.3% vs. 54.7%, P = 0.016), improved renal function recovery rates (60.6% vs. 53.7%), and shortened hospital stay and mechanical ventilation duration (Gordon et al., 2016). However, the IDEAL-ICU trial enrolled 600 critically ill patients, including 488 with sepsis or AKI, and showed that early initiation of CRRT (within 12 h after reaching KDIGO Stage 3 AKI) was associated with higher mortality than delayed initiation (59% vs. 54%), suggesting that early initiation may pose other risks (Hellman et al., 2021; Tandukar and Palevsky, 2019). Both trials emphasize the importance of early intervention, but the results differ: the ELAIN trial supports early initiation of CRRT, while the IDEAL-ICU trial suggests that delayed initiation may be more beneficial, which may be related to patient severity and treatment strategies.

These study results have important implications for clinical practice. In patients with severe AKI, especially those meeting KDIGO Stage 2 criteria, early initiation of CRRT may be more effective (Gaudry et al., 2022; Zarbock et al., 2016); however, in patients with sepsis-associated AKI, early initiation may not always be the best choice, and individualized decisions should be made based on the patient’s specific condition (Hellman et al., 2021; Ostermann et al., 2016).

#### 4.3.2 Immune adsorption potential of novel filter membrane materials (e.g., AN69Oxiris)

Advancements in filter membrane materials have provided new possibilities for optimizing CRRT. For example, the AN69Oxiris filter membrane exhibits excellent biocompatibility and immune adsorption capabilities, effectively removing inflammatory mediators from the blood (Zang et al., 2022; Kim et al., 2024). The application of this filter membrane material not only improves renal function but may also have a positive effect on the control of systemic inflammatory response syndrome (SIRS) (Kim et al., 2024).

### 4.4 Exploration of targeted therapies

Inflammatory responses play a crucial role in the occurrence and development of sepsis and AKI. Therefore, regulating inflammatory responses has become an important direction for treatment. Recombinant human alkaline phosphatase (recAP) is a novel anti-inflammatory drug that has demonstrated good safety and efficacy in Phase II clinical trials (Ostermann et al., 2016). Studies have shown that recAP can significantly reduce serum inflammatory mediator levels and improve patient prognosis (Tunjungputri et al., 2016).

Mitochondrial dysfunction is one of the important pathophysiological mechanisms of sepsis and AKI. Mitochondrial protectants (such as SS31) and iron chelators (such as deferoxamine) have shown protective effects on the kidney in preclinical studies (Yu et al., 2023; Liu D. et al., 2020; Sun et al., 2025; Fraga et al., 2016). These drugs protect mitochondrial function by reducing oxidative stress and iron overload, thereby alleviating renal injury.

## 5 Prognosis and long-term impacts

### 5.1 Short-term prognosis prediction models: machine learning-based stratification systems for SAAKI

In the management of SAAKI, accurately predicting patients’ short-term prognosis is crucial for optimizing treatment strategies and improving patient outcomes. Recently, machine learning (ML) technology has been extensively applied in the medical field, particularly in predicting disease prognosis. ML-based stratification systems for SAAKI, such as AIAPACHE (Artificial Intelligence Acute Physiology and Chronic Health Evaluation), leverage a multitude of clinical data to provide a more precise assessment of patients’ short-term risks.

By integrating physiological parameters, laboratory test results, medical histories, and other multidimensional data, the AIAPACHE system employs sophisticated algorithmic models to stratify patients into risk categories. Studies have demonstrated that such ML-based models excel in predicting the short-term prognosis of SAAKI patients, with prediction accuracy surpassing traditional scoring systems (Li X. et al., 2023; Luo et al., 2021; Li et al., 2024). These models empower clinicians to identify high-risk patients early, enabling timely interventions to enhance patients’ short-term prognosis.

### 5.2 Long-term renal outcomes

The long-term prognosis of SAAKI patients is influenced by various factors. A recent cohort study revealed that the incidence of chronic kidney disease (CKD) among SAAKI patients within 1 year post-discharge is three times higher than that of non-SAAKI patients. Furthermore, SAAKI patients exhibit a significantly increased risk of progressing to end-stage renal disease (ESRD) compared to non-SAAKI patients (Lee et al., 2022; Tao et al., 2021). The transition from acute kidney injury to CKD in sepsis survivors is a chronic process requiring a 1-year follow-up study to assess its impact. Notably, sepsis-induced acute kidney injury is intimately linked to long-term mortality and CKD (Lee et al., 2022). Biomarkers such as extracellular DNA (ExoDNA) and mitochondrial DNA have been extensively studied in sepsis, with ExoDNA predictive of 28-day mortality and long-term prognosis, while mitochondrial DNA correlates with immunosuppression and prognosis (Dennhardt et al., 2024). A multinational, multicenter study found that 5%–6% of intensive care unit (ICU) patients experience acute renal failure (ARF), with poor renal function recovery and high dependency levels post-discharge (Uchino et al., 2005). Management strategies for SAAKI emphasize the importance of hemodynamic and blood transfusion protocols, with maintaining higher mean arterial pressure deemed crucial for improving outcomes in septic shock (Maheshwari et al., 2018; Ruan et al., 2023). The advent of multiomics technologies offers new perspectives for research, enabling a better understanding of pathophysiology and diagnostic biomarkers through the integration of data from renal tissue, blood, and urine samples, thereby facilitating personalized diagnosis and clinical decision-making (Qiao and Cui, 2022).

Research also indicates that the speed and extent of renal function recovery vary among SAAKI patients during long-term follow-up post-discharge. Approximately 20%–50% of SAAKI patients experience continued decline in renal function, ultimately progressing to CKD. Advanced age, poor baseline renal function, multiple comorbidities, and severity of AKI are identified as high-risk factors for the progression to CKD among SAAKI patients (Lee et al., 2022; Tao et al., 2021).

### 5.3 Multi-organ interactions

The reno-cerebral axis, which describes the interaction between the kidneys and the brain, is particularly significant in SAAKI patients (Mao et al., 2020). Acute decline in renal function leads to the accumulation of toxins in the body, subsequently affecting brain function (Chen X. et al., 2024). Studies have shown that the incidence of cognitive dysfunction and neuropsychiatric symptoms significantly increases in SAAKI patients post-acute phase (Ramírez-Guerrero et al., 2023). Long-term follow-up reveals that SAAKI patients are twice as likely to experience cognitive decline within 1 year post-discharge compared to non-SAAKI patients. Additionally, damage to the reno-cerebral axis may increase the risk of long-term neurodegenerative diseases. For instance, SAAKI patients are 1.5 times more likely to develop Alzheimer’s disease within 5 years post-discharge. This long-term neurological damage may be associated with chronic inflammation and oxidative stress resulting from renal insufficiency (Poston and Koyner, 2019; Liu et al., 2022).

The reno-intestinal axis refers to the interaction between the kidneys and the intestines (Liang et al., 2024). SAAKI patients often experience intestinal dysfunction during the acute phase, which may persist post-discharge and impact long-term prognosis (Foresto-Neto et al., 2021). Intestinal barrier dysfunction is a hallmark of sepsis, leading to bacterial translocation and endotoxin entry into the bloodstream, further exacerbating systemic inflammatory responses (Jia et al., 2024). This not only affects the kidneys but may also cause damage to other organs (Ge et al., 2020). For example, the role of IL-17A in sepsis extends beyond the kidneys, involving intestinal barrier dysfunction by inhibiting intestinal epithelial cell proliferation and inducing apoptosis, resulting in intestinal bacterial translocation (Ge et al., 2020). Studies show that the incidence of intestinal infections and inflammatory bowel disease within 1 year post-discharge is significantly higher in SAAKI patients compared to non-SAAKI patients (Xu Y. et al., 2024). Furthermore, damage to the reno-intestinal axis may lead to long-term nutrient malabsorption and immune dysfunction. Long-term follow-up reveals that SAAKI patients are twice as likely to experience malnutrition and immune deficiency within 3 years post-discharge compared to non-SAAKI patients. This long-term intestinal dysfunction may be related to chronic inflammation and gut microbiota dysregulation resulting from renal insufficiency (Poston and Koyner, 2019; Liu et al., 2022).

## 6 Challenges and future directions

### 6.1 Fundamental research bottlenecks

Fundamental research has played a pivotal role in exploring the pathophysiological mechanisms of SAAKI, but pathological differences between humans and animal models remain a significant challenge. For instance, rodent models are widely used in research, but their renal structure differs significantly from humans, particularly in the absence of cortical nephrons in rodents. Moreover, significant differences exist in the immune microenvironment between animal models and humans, which may affect the universality of research findings (Hulst et al., 2024; Xing et al., 2018). Additionally, experimental designs and induction methods in animal models (such as LPS injection) may not fully mimic the pathophysiological processes of human septic AKI (Hulst et al., 2024), leading to observed pathological changes and treatment effects in animal models that cannot be fully translated into human clinical practice, thereby limiting the translation of fundamental research results into clinical treatment (IntechOpen, 2022).

### 6.2 Translational medicine opportunities

The development of single-cell sequencing technology has brought new opportunities for SAAKI research. Through single-cell sequencing, detailed analysis of renal cell heterogeneity can be conducted, revealing the roles of different cell types in SAAKI (Tang et al., 2021; Atreya et al., 2023). This technology aids in better understanding the cellular mechanisms of renal injury and provides a basis for developing targeted treatments. For example, single-cell sequencing can identify specific cell subpopulations that play crucial roles in SAAKI, thereby providing potential targets for precision therapy (Li et al., 2021b).

Organ-on-a-chip technology, an emerging *in vitro* model technology, simulates the microenvironment of human organs on a chip. This technology can be used to study the pathophysiological mechanisms of SAAKI and test potential treatments (Huang et al., 2024). Organ-on-a-chip simulates renal blood flow, oxygen supply, and cell-cell interactions, providing a more precise platform for studying the microenvironment of SAAKI. Compared to traditional animal models, organ-on-a-chip technology better simulates the physiological and pathological states of human kidneys, promising to become an important tool for future SAAKI research.

### 6.3 Precision medicine prospects

The core of precision medicine lies in developing personalized treatment plans based on individual patient characteristics. In the treatment of SAAKI, the concept of endophenotypes is gradually gaining attention. Endophenotypes refer to disease subtypes based on pathophysiological mechanisms. By identifying different endophenotypes of SAAKI, more precise treatment can be provided for patients. For example, SAAKI patients can be classified into different endophenotypes based on factors such as the intensity of inflammatory responses, types and severity of renal injury, thereby allowing the development of the most suitable treatment plan for each patient (Li et al., 2021b).

Biomarkers play a crucial role in the diagnosis and treatment of SAAKI. By monitoring changes in biomarkers, the extent of renal injury and treatment effectiveness can be assessed in real-time, enabling dynamic adjustments to treatment plans. For instance, novel biomarkers such as neutrophil gelatinase-associated lipocalin (NGAL), kidney injury molecule-1 (KIM-1), and interleukin-18 (IL-18) can provide early indications of renal injury onset and progression. By utilizing these biomarkers, treatment plans can be timely adjusted to enhance treatment effectiveness (Poston and Koyner, 2019; Zarbock et al., 2023a; Zarbock et al., 2023b; Kounatidis et al., 2024; Xu J. et al., 2023; Xu D. et al., 2024).

Research on SAAKI faces various challenges and opportunities, including fundamental research bottlenecks, translational medicine opportunities, and precision medicine prospects. The pathological differences between human and animal models limit the clinical translation of research findings, but the development of single-cell sequencing and organ-on-a-chip technologies offers new solutions to this problem. The promise of precision medicine lies in individualized treatment guided by endophenotypes and dynamic treatment adjustments driven by biomarkers, improving SAAKI treatment outcomes. Future research needs to further explore these areas, driving SAAKI treatment towards precision and personalization.

## 7 Conclusion

SAAKI is one of the most common severe complications in sepsis patients, with high morbidity and mortality rates and significant long-term health impacts. This paper reviews the latest advancements in the pathophysiological mechanisms, diagnostic progress, treatment strategies, and prognosis research of SAAKI. In terms of pathophysiological mechanisms, research has shifted from the traditional renal ischemia-centric view to multidimensional interactions such as microcirculatory disturbances, immune metabolic disorders, and programmed cell death, revealing the pivotal roles of pyroptosis, NETs-mediated damage, and mitochondrial dysfunction and metabolic reprogramming in SAAKI. Diagnostically, traditional KDIGO criteria exhibit limitations, whereas novel biomarkers (e.g., NGAL, KIM-1, IL-18) and imaging techniques (e.g., renal ultrasound shear wave elastography) offer new tools for early diagnosis and disease monitoring. With regard to treatment strategies, early intervention, hemodynamic management, renal replacement therapy, and targeted therapy are the mainstay approaches, albeit with ongoing controversies regarding initiation timing and methodologies. Prognostic research focuses on mechanisms underlying the transition from SAAKI to CKD and preventive strategies, emphasizing the impact of multi-organ interactions on patients’ long-term prognosis.

Nonetheless, a “cognition-practice gap” persists in the diagnosis and treatment of SAAKI. Future research must bridge the pathological differences between animal models and humans and explore the potential of multiomics technologies and artificial intelligence in optimizing management, thereby advancing SAAKI treatment towards precision and personalization. By integrating these advancements, we can strive to mitigate the burden of SAAKI, enhance patient outcomes, and ultimately contribute to a deeper understanding and more effective management of this complex and devastating condition.
